# Analysis and Design of a Polygonal Oblique Beam for the Butterfly Vibratory Gyroscope with Improved Robustness to Fabrication Imperfections

**DOI:** 10.3390/mi9050198

**Published:** 2018-04-24

**Authors:** Fenlan Ou, Zhanqiang Hou, Xuezhong Wu, Dingbang Xiao

**Affiliations:** College of Intelligence Science and Engineering, National University of Defense Technology, Changsha 410073, China; oufenlan15@nudt.edu.cn (F.O.); xzwu@nudt.edu.cn (X.W.); dingbangxiao@nudt.edu.cn (D.X.)

**Keywords:** gyroscope, spindle azimuth angle, fabrication imperfections, tolerant capability, quadrature error

## Abstract

This paper focuses on structural optimization of a Butterfly vibratory gyroscope (BFVG). An oblique suspension beam adopting polygonal cross-section is proposed in order to enhance the sensitivity and robustness. The operation principles of the BFVG are introduced. The suspension beam, which was found to be the key component, is selectively stressed. Varying cross sections of the suspension beam, including parallelogram, pentagon, hexagon, platform of pentagon, L-shaped and convex shapes are compared with each other. In particular, in order to show the advantages of the proposed polygonal cross-section, the convex cross-section is used as a reference. The influence of fabrication imperfections, which includes alignment error, silicon thickness error, etching depth error, upper width error, bottom width error and deep reactive-ion etching (DRIE) verticality error, on the oblique beam’s spindle azimuth angle of the two cross-sections is analyzed. Further, the quadrature error of two cross-sections with a fabrication defect is analyzed. The theoretical arithmetic results suggest that a polygonal cross-section beam is much more stable than a convex cross-section beam in most cases. The robustness of the fabrication imperfection is improved nine-fold and the quadrature error due to fabrication defect is reduced by 70 percent with a polygonal cross-section. It could be a better candidate for BFVG’s oblique beam, which would provide a gyroscope with good robustness and repeatability.

## 1. Introduction

A gyroscope is a sensor that measures the angular motion of a vehicle. It is the core device in inertial navigation systems. Similar to most other Micro-Electro-Mechanism System (MEMS) applications, the key for success within the area of inertial gyroscopes is the comprehensive development and integration of all of the components necessary for these multidisciplinary products [1]. High-performance angular rate sensors, such as precision fiber-optic gyroscopes, ring laser gyroscopes, and conventional rotating wheel gyroscopes are usually too expensive and too large for use in most emerging applications [2,3,4,5,6,7]. Compared with these, the major advantages of micro-machined vibratory gyroscopes include miniaturization, batch fabrication, low-cost, high reliability and integrated circuit (IC) compatible integration [8,9]. Micro-machined vibratory gyroscopes are important components in inertial navigation systems, which are widely used in both military and civil fields [10,11]. However, in the market of tactical and inertial grade gyroscopes, proving reliable devices with long-term stability remains probably the greatest challenge.

Electrostatic driving and capacitive detection are the most widely-used and successful operation modes for gyroscopes. The main types of gyroscope are the comb gyroscope and the plate capacitor gyroscope. The comb gyroscope is driven and detected by the reciprocating motion of toothed electrodes. The small electrode area is a disadvantage of this gyroscope. It cannot obtain a large signal to detect capacitance change and the fabrication process of the tooth structure is very complicated [12,13,14,15]. The plate capacitor gyroscope improves the shortcoming in the detection area. However, the ordinary plate capacitor gyroscope can only achieve in-plane displacement, and its driving and sensing electrodes should be placed vertically. This undoubtedly increases the difficulty of the design and manufacture [16,17,18]. The butterfly vibratory gyroscope (BFVG) is classified as one kind of high sensitivity parallel plate capacitor gyroscope due to its unique structure. In addition, it has been widely-used and studied [19,20,21]. The BFVG shows superiority in size, accuracy, operation life and performance. It measures angular velocity via capacitive sensing, where displacement arises as a result of the Coriolis Effect [22,23]. The performance of this devices is strongly dependent on the sensitivity of its capacitors. It is simple to manufacture with single-sided electrostatic excitation and capacitive detection [24,25,26]. The most important part of the BFVG is the design of the oblique beam. Since the oblique beam is incline, the bending moment is generated by applying force on the plane of the BFVG. In this way, the four proof-masse can vibrate under the driving mode. The driving electrode and the sensing electrode can be made in one plane, which simplifies the fabrication process of the micro-gyroscope greatly. Also, the stability of the gyroscope is improved. 

In BFVGs, the oblique beam is the most important part of the structure. By changing the inclined cutting position, we can achieve different spindle azimuth angles on different oblique beam’s cross-sections. Every dimension of the oblique beam can affect the beam’s spindle azimuth angle. In the fabrication process of a gyroscope, fabrication imperfections are bound to occur. Thus, the selection of a suitable spindle azimuth angle and the discussion around the tolerance capability of the oblique beam’s cross-section are two key problems of this study. Currently, many studies have been done on the cross-section of the BFVG oblique beam. 

The Imego AB (Gothenburg, Sweden) institute in Sweden and the National University of Defense Technology (NUDT) have carried out a long-term study of the parallelogram oblique beam of BFVG [27,28,29,30]. The anisotropic wet etching (AWE) process has been adopted in this manufacturing technology process to fabricate the parallelogram oblique beam. The cross-section of the oblique beam is formed by (100) and (111) crystal planes with an angle of 54.74°. This design has great limitations in the spindle azimuth angle [27]. In order to improve the performance of the gyroscope, NUDT presented a new oblique BFVG beam type. The hexagonal oblique beam is produced using precision, time-controlled multilayer pre-buried masks. This design can increase the spindle azimuth angle to reduce the mismatch between the excitation and detection frequencies. However, it is still difficult to achieve a large angular range, and it is hard to guarantee the symmetry of the beam [31,32]. In order to be able to flexibly adjust the spindle azimuth angle, the following cross-sections are proposed. The Sensonor Company in Norway adopted AWE and DRIE-combined fabrication technology to produce the cross-section of a gyroscope in 2001. The AWE was used to give one corner of the beam an extra recess prior to the DRIE to release the structure [8]. In addition, they proposed an L-shaped cross-section by using the DRIE to achieve an incline in the oblique beam. The NUDT presented a pentagon cross-section with AWE and DRIE-combined fabrication technology [33]. In this design, the spindle azimuth angle of the oblique beam can be flexibly changed by adjusting the width of the etching dimension. However, due to the inconsistent etching depth on both sides of the oblique beam, the internal etching occurs on the asymmetric side. This will affect the spindle azimuth angle and the performance of the gyroscope. Therefore, the cross-section of the gyroscope is optimized from the point of view of the fabrication process. Later in 2010, the Sensonor Company designed a micro-gyroscope with DRIE. The beams, all having identical cross-sectional geometry, exhibited a convex oblique beam. The shape of the convex cross-section beam was obtained by two DRIE process [34]. Although this design can reduce the internal etching effect on the oblique beam, the tolerance for fabrication imperfections is unsatisfactory. So, the design of a gyroscope cross-section which has a low sensitivity to fabrication imperfection is the key to research. 

In this paper, we demonstrate that structural design can influence the robustness of a gyroscope’s structure and effectively reduce the influence of fabrication imperfection on structural performance. In the first section, the research background is introduced. In the second section, the operating principles and theoretical analysis of the BFVG are introduced. The robustness of a convex cross-section is analyzed. In addition, the polygonal cross-section is proposed. In the third section, a comparison between the two cross-sections is made, and the types of fabrication imperfection used for comparison are put forward. In the fourth section, the tolerance capability of the two cross-sections are analyzed. In the last section, the quadrature error caused by fabrication defect is compared between two cross-sections. In conclusion, we confirm that the polygonal cross-section shows good robustness. That can give the BFVG has stronger stability and reach the goal of optimization design. 

## 2. Structural Design and Theoretical Analysis

The BFVG is one kind of capacitive micro-machined vibratory gyroscope based on the Coriolis effect. The basic architecture of a vibratory gyroscope comprises a driving mode oscillator that generates and maintains a constant linear or angular momentum, coupled to a sensing mode that measures the sinusoidal Coriolis force induced due to the combination of the drive vibration and an angular rate input [20].

### 2.1. The Operating Principles of the Gyroscope

The structure of the BFVG includes the sensitive structure and the electrode structure. The sensitive structure of the BFVG consists of a four proof-mass, two cantilever beams, one oblique beam and two anchors. The electrode structure mainly includes two driving electrodes and a sensing capacitive electrode. There is an electrode gap between the sensitive structure and electrode structure after bonding. 

**Figure 1:**
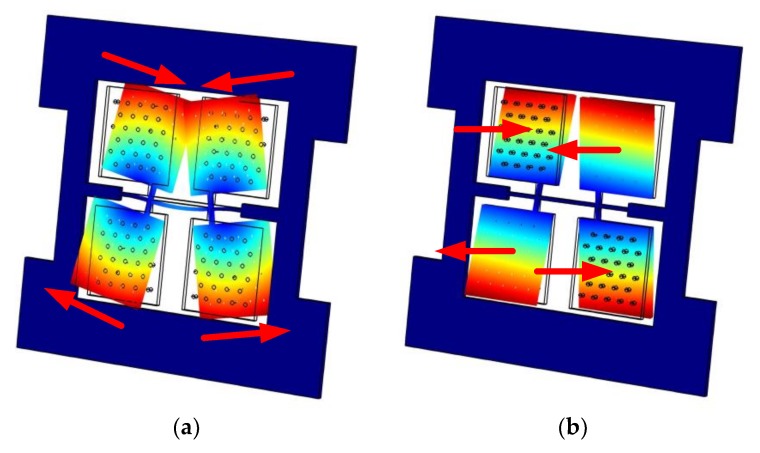
The operation modes of the butterfly vibratory gyroscope (BFVG) by COMSOL (5.2, Stockholm, Sweden). (**a**) The driving mode; (**b**) The sensing mode.

The BFVG has two operating modes: the driving mode and the sensing mode. Both of the drive and sense resonators are modeled as a mass-spring-damper system [21]. Since the Coriolis effect is based on the conservation of momentum, the gyroscope requires a mechanical oscillator to generate momentum, which is called the driving mode oscillator. The driving mode oscillator operates on self-resonance and achieves a stable amplitude and phase by the use of the Phase Lock Loop and Automatic Gain Control methods. The driving mode involves the bending vibration of the oblique beam. The motion form is shown in Figure 1a. The sensing mode is designed to detect the Coriolis effect. When an angular rate is input, there will be a sinusoidal oscillation in the detection direction due to the Coriolis force. The sensing mode involves the torsional vibration of the oblique beam. The motion form is shown in Figure 1b. 

### 2.2. Structural Design and Operation Principles

**Figure 2:**
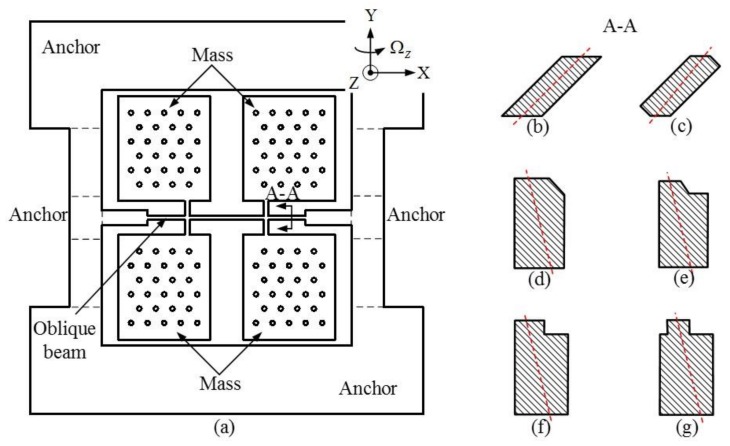
Vibratory structure of the BFVG. (**a**) The prototype of the gyroscope; (**b**) An oblique beam with a parallelogram cross-section; (**c**) An oblique beam with a hexagonal cross-section; (**d**) An oblique beam with a pentagon cross-section; (**e**) An oblique beam with a platform of pentagon cross-section; (**f**) An oblique beam with an L-shaped cross-section; (**g**) An oblique beam with a convex cross-section.

The reliability and sensitivity of micro-machined inertial sensors, such as gyroscopes, are primarily determined by their structural properties. The structure of the BFVG consists of a four proof-mass, two cantilever beams, one oblique beam and two anchors. The structure is shown in Figure 2a. According to the existence of the oblique beam, the driving and sensing forces of BFVG are determined by the spindle azimuth angle. It is important to design a strong, robust oblique beam cross-section which is flexible enough to be regulated at the spindle azimuth angle. Currently, many different types of oblique beam cross-section have been proposed in domestic and oversea research of BFVG. Figure 2b–g show the shapes of various cross-sections: parallelogram cross-section, parallel-piped cross-section, pentagon cross-section, pentagon step cross-section, L-shaped cross-section and convex cross-section. The red dashed line is the inertial spindle of the cross-sections. The spindle azimuth angle is one of the most important factors that affect the drive efficiency of a gyroscope. Thus, whatever the shape of the beam’s cross-section, the value of the spindle azimuth angle is the key factor that affects the performance of the gyroscope.

**Figure 3:**
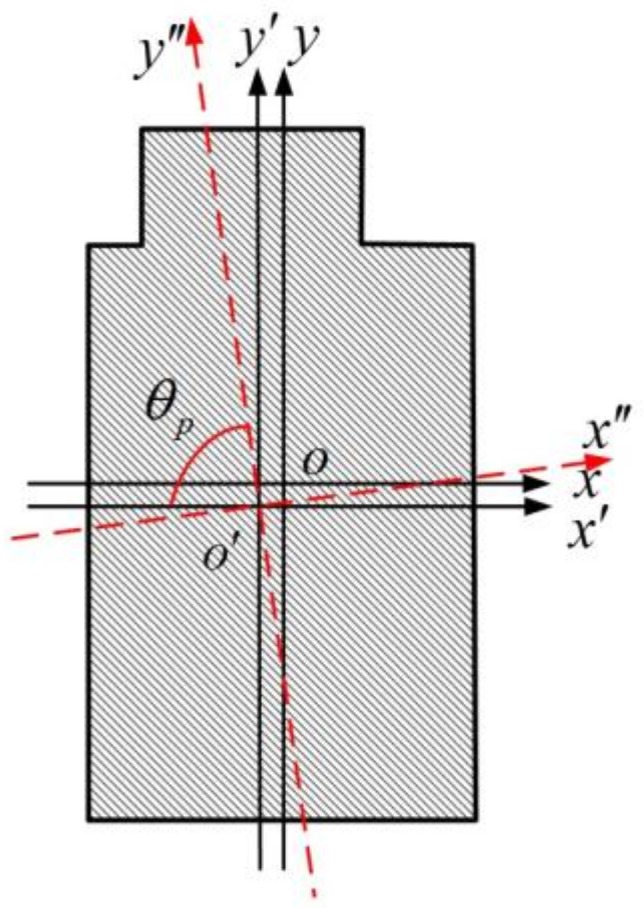
Cross-sectional view of the oblique beam in the Sensonor Company’s gyroscope.

Here, we present the convex cross-section of the Sensonor Company’s (Horten, Norway) new product as an example. It is shown in Figure 3. The original axes of the rectangular cross-section are *xoy* and *o* is the center of the rectangle. When there is an unfilled corner in the rectangular cross-section, the axes deviate to *x*′*o*′*y*′. *x*″ and *y*″ are the inertia axes of the cross-section. The angle between *x*′ and *y*″ is the cross-section’s spindle azimuth angle. We use *θ_p_* to represent it.

The operation modes of BFVGs are the driving mode and the sensing mode. The *X*-axis is the driving direction, the *Y*-axis is the sensing direction, and the *Z*-axis is sensitive to the direction of angular acceleration. There is a silicon electrode under each mass to measure the driving and sensing capacitances. The structural parameters, derived from a large number of simulations and theoretical calculations, are listed in Table 1.

### 2.3. Spindle Azimuth Angle

In this section, we focus on the spindle azimuth angle’s effect on the gyroscope’s performance, when a convex cross-section is used (this has been studied by Sensonor Company recently). Subsequently, we choose the optimum value for the spindle azimuth angle to improve the performance of the gyroscope.

The formulas to calculate the spindle azimuth angle are as follows:

The inertial matrix for the *x* axis is solved as

(1) $$I_{x}=\int_{A}y^{2}dA,$$

The inertial matrix for the *y* axis is solved as

(2) $$I_{y}=\int_{A}x^{2}dA,$$

And the product of inertial of the section to the origin point *o* is solved as

(3) $$I_{xy}=\int_{A}xydA.$$

According to the parallel axis theorem, we can move the coordinate system to $x^{\prime }$. The following formula can be obtained:

(4) $$\begin{array}{l} I_{x^{\prime }}=I_{x}-A{\bar{x}}^{2}\\ I_{y^{\prime }}=I_{y}-A{\bar{y}}^{2}\\ I_{x^{\prime }y^{\prime }}=I_{xy}-A\bar{x}\cdot \bar{y} \end{array}$$

where $\bar{x}$ and $\bar{y}$ represent the position of the centroid in the coordinate system, *OXY*.

$I_{x^{\prime }}$ and $I_{y^{\prime }}$ are the moments of inertia, which are the centroid offsets for axes $x^{\prime }$ and $y^{\prime }$ of the oblique beam’s cross-section.

A, the area of the oblique beam’s cross-section.

The spindle azimuth angle of the oblique beam can be given by

(5) $$\theta_{p}=\frac{\pi }{2}-\frac{\arctan\left(\frac{-2I_{x^{\prime }y^{\prime }}}{I_{x^{\prime }}-I_{y^{\prime }}}\right)}{2}.$$

The spindle azimuth angle of any cross-section of an asymmetric beam can be calculated by this formulae. Any change in the shape and size of the oblique beam will have a big influence on the characteristics of a gyroscope, including the frequency, frequency mismatch, stiffness and sensitivity. COMSOL Multiphysics 5.2 simulation and theoretical derivation were carried out to analyze and verify the relationship between the spindle azimuth angle of the oblique beam and the sensitivity of the gyroscope.

### 2.4. Capacitance Sensitivity

The BFVG is excited by electrostatic force. Its dynamic governing equations can be built from the Coriolis Effect and Newton’s second law. The dynamical equation can be simplified as

(6) $$\left\{ \begin{array}{l} I_{d}{\ddot{\phi }}_{d}+I_{d}\frac{\omega_{d}}{Q_{d}}{\dot{\phi }}_{d}+I_{d}\omega_{d}^{2}\phi_{d}=k_{cd}V_{dc}V_{ac}\cos\theta_{p}\sin\omega_{d}t\\ I_{s}{\ddot{\phi }}_{s}+I_{s}\frac{\omega_{s}}{Q_{s}}{\dot{\phi }}_{s}+I_{s}\omega_{s}^{2}\phi_{s}=-2\Omega {\dot{\phi }}_{d}I_{s}\sin\theta_{p} \end{array}\right.$$

where the coefficients are represented by

$\dot{\phi }$, the related angular velocities of the rotations;
$\ddot{\phi }$, the angular accelerations of the rotations;
$I_{d}$, the moments of inertia of the bending axis;
$I_{s}$, the moments of inertia of the torsion axis;
$\omega_{d}$, the resonant frequency of the driving mode;
$\omega_{s}$, the resonant frequency of the sensing mode.
$Q_{d}$, the related quality factors of the driving mode;
$Q_{s}$, the related quality factors of the sensing mode;
Ω, the angle rate input;
$V_{dc}$, the amplitude of the direct voltage;
$V_{ac}$, the amplitude of the alternating voltage; and
$k_{cd}$, the velocity coupling coefficient. The formula can be expressed as
  (7) $$k_{cd}=\frac{4\varepsilon \varepsilon_{0}\omega_{d}l_{d}(\omega_{d}+\omega_{b})}{d^{2}}.$$

Here, ε, the relative dielectric constant, and $\varepsilon_{0}$, the dielectric constant of air.

$l_{d}$, the length of the driving electrode’s area.

d, the interval between the sensitive structure and the electrode, which can simply represent an electrode gap.

The angular vibrations under the driving mode are solved with

(8) $$\phi_{d}=\frac{k_{cd}V_{dc}V_{ac}Q_{d}\cos\theta_{p}}{I_{d}\omega_{d}^{2}}.$$

From the formula, we obtain the vertical displacement and driving amplitude when the gyroscope is vibrating under driving mode. They are referred to as the out-of-plane displacement (*D_z_*) and in-plane displacement (*D_x_*), respectively. Here, *d_x_* is the distance from the location of the maximum structure displacement to the center of the beam. *d_z_* is the distance from the location of the maximum structure displacement to the center of mass:

(9) $$D_{z}=\phi_{d}\cos\theta_{p}d_{z}$$

(10) $$D_{x}=\phi_{d}\sin\theta_{p}d_{x}.$$

The driving capacitance change is expressed by formula (11). *A_d_* is the driving electrode area of the gyroscope:

(11) $$D_{cd}=\frac{4\varepsilon_{0}A_{d}V_{dc}V_{ac}k_{cd}\cos\theta_{p}Q_{d}}{d^{2}I_{d}\omega_{d}{}^{2}-dV_{dc}V_{ac}k_{cd}\cos\theta_{p}Q_{d}}.$$

Substituting formula (7) into formula (6), we get the differential equation for the sensing mode of a BFVG:

(12) $$\phi_{s}=\frac{2\Omega \sin\theta_{p}\phi_{d0}\omega_{d}}{\sqrt{{\left(\omega_{s}^{2}-\omega_{d}^{2}\right)}^{2}+{\left(\frac{\omega_{d}\omega_{s}}{Q_{s}}\right)}^{2}}}\sin\left(\omega_{d}t+\varphi \right)$$

(13) $$\varphi =-\tan^{-1}\frac{\omega_{d}\omega_{s}}{Q_{s}\left(\omega_{s}^{2}-\omega_{d}^{2}\right)}.$$

After synchronous demodulation by the sinusoidal voltage at the mechanical resonance frequency and low pass filter, the output of the angular rate is solved as

(14) $$V_{out}=\frac{k_{cs}\sin\theta_{p}\phi_{d0}\omega_{d}}{\sqrt{{\left(\omega_{s}^{2}-\omega_{d}^{2}\right)}^{2}+{\left(\frac{\omega_{d}\omega_{s}}{Q_{s}}\right)}^{2}}}\cos\varphi \cdot \Omega$$

$k_{cs}$, the velocity coupling coefficient.

(15) $$k_{cs}=\frac{2\varepsilon \varepsilon_{0}\omega_{s}l_{s}(l_{s}+l_{b})}{d^{2}}.$$

The analytical model of the capacitance sensitivity is expressed as

(16) $$S_{g}=\frac{V_{out}}{\Omega }=\frac{k_{cd}k_{cs}V_{dc}V_{ac}Q_{d}\sin2\theta_{p}}{2I_{d}\omega_{d}\sqrt{{\left(\omega_{s}^{2}-\omega_{d}^{2}\right)}^{2}+{\left(\frac{\omega_{d}\omega_{s}}{Q_{s}}\right)}^{2}}}\cos\varphi .$$

As above, the BFVG adopts electrostatic force to achieve large amplitude motion of the mass block in the horizontal direction. The sensing mode is motivated by the input of angle velocity. The capacitance change between the sensitive structure and the electrode structure is detected to determine the angle velocity. The theoretical analysis shows that the electrode gap and the spindle azimuth angle are both significant, key parameters that affect the performance of BFVG. What is more, they are mutually related and constrained. Under this condition, the influence of the spindle azimuth angle on a gyroscope’s capacitance sensitivity is discussed in this paper, so the electrode gap of gyroscope is assumed to be fixed.

The BFVG model of analysis was mentioned earlier. Due to the existence of the oblique beam, the bending moment has out-of-plane displacement and in-plane displacement components under the driving mode. Because the value of the electrode gap is fixed, the ultimate value of the in-plane displacement is fixed as ($D_{z}=d(\max)$). 

Using formula (8) and formula (9), the relationship between driving voltage and spindle azimuth angle can be deduced as follows:

(17) $$V_{dc}V_{ac}=\frac{D_{z}I_{d}\omega_{d}^{2}}{k_{cd}Q_{d}\cos^{2}\theta_{p}d_{z}}.$$

**Figure 4:**
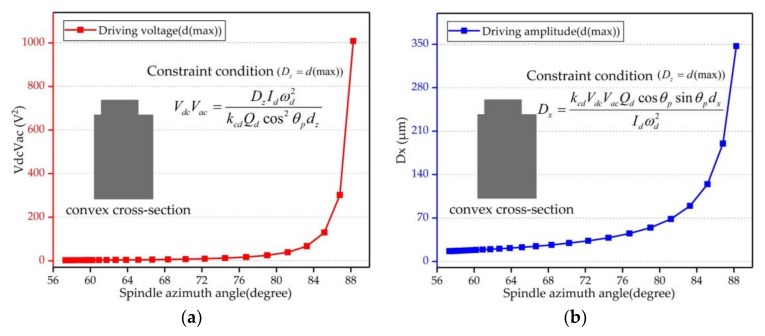
Driving voltage and driving amplitude of the convex cross-section beam, when the in-plane displacement was fixed at d(max); (**a**) shows the required driving voltages of different spindle azimuth angles; (**b**) shows the corresponding driving amplitudes of different spindle azimuth angles.

If we assume the in-plane displacement has achieved its ultimate value, we can obtain the driving voltages of different spindle azimuth angles and the maximum out-of-plane displacement under these driving voltages. The pattern of change is shown in Figure 4 with a block point curve. Therefore, the BFVG had a large bending angle when the spindle azimuth angle was in the range of 56° to 72°. It did not need too much driving voltage to achieve the ultimate value of in-plane displacement. As a result, the out-of-plane displacement cannot be improved, and the sensitivity of the gyroscope is limited. So, the spindle azimuth angle should be increased. However, based on the dates of analysis, the driving voltage exceeded the voltage provided by the analog circuit when the spindle azimuth angle was raised. Further, the value of the corresponding driving amplitude was not reasonable.

**Figure 5:**
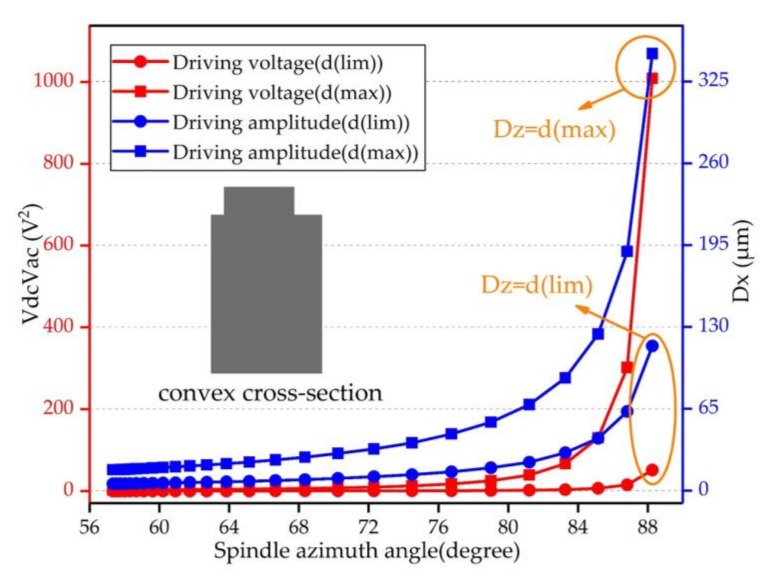
Driving voltage and driving amplitude of the convex cross-section beam—this figure has two *Y* axes. The ultimate in-plane displacement of the driving mode is fixed, the spindle azimuth is changing. The left axis shows the voltage required to achieve the ultimate in-plane displacement of the driving mode under different spindle azimuth angles. The right axis shows the corresponding ultimate driving amplitude of the gyroscope.

Therefore, according to the above analysis, it is necessary to consider the electrode gap error due to the fabrication process, the material pull-in effect and the nonlinear effect. We designed the gyroscope’s ultimate in-plane displacement under the driving mode reasonably and set *d*(lim) as the ultimate value for different spindle azimuth angles. The data is shown in Figure 5 with a dotted point curve. The analysis dates show that the driving voltage and the driving amplitude values were reasonable and did not produce the pull-in effect.

Substituting formula (10) into formula (16), we know that if we want to improve the sensitivity of the BFVG under the condition of a fixed electrode gap, we need to improve the driving amplitude of the gyroscope:

(18) $$S_{g}=\frac{2\varepsilon \varepsilon_{0}\omega_{s}l_{s}(l_{s}+l_{b})D_{x}\omega_{d}}{d^{2}d_{x}\sqrt{{\left(\omega_{s}^{2}-\omega_{d}^{2}\right)}^{2}+{\left(\frac{\omega_{d}\omega_{s}}{Q_{s}}\right)}^{2}}}\cos\varphi .$$

**Figure 6:**
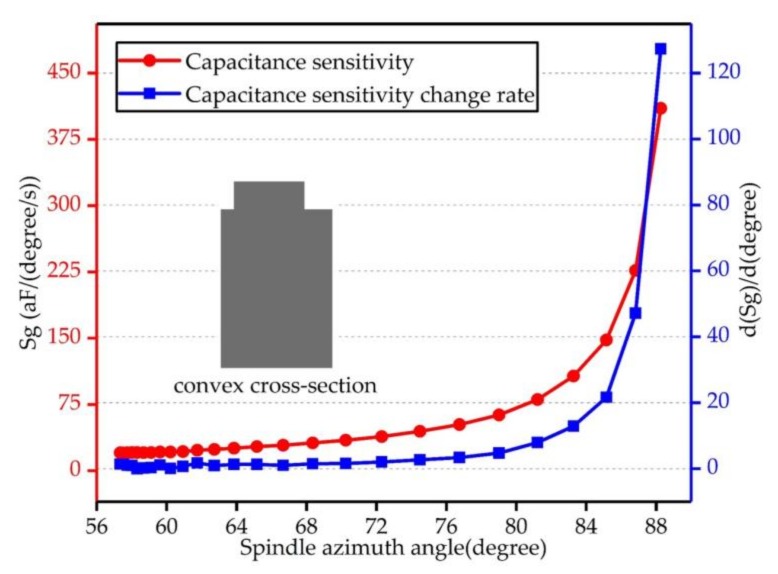
Capacitance sensitivity and rate of capacitance sensitivity change for the convex cross-section beam—the ultimate in-plane displacement of driving mode is fixed and the driving amplitude is maximal. This figure shows the maximum value of the capacitance sensitivity when the gyroscope has different spindle azimuth angles. The right vertical axis is the rate of capacitance sensitivity change.

Furthermore, under the ultimate driving amplitude value in the BFVG's driving mode, the rates of capacitance sensitivity and capacitance sensitivity change for the different spindle azimuth angles were also compared and analyzed. The relation curves are shown in Figure 6; the horizontal axis of Figure 6 is the range of the gyroscope’s spindle azimuth angle. The left and right axes are the capacitance sensitivity and the change in capacitance sensitivity of the gyroscope with convex cross-section, respectively. The figure shows that, when the spindle azimuth angle ranged from 56° to 80°, the capacitance sensitivity changed slowly. When the spindle azimuth angle was bigger than 80°, the capacitance sensitivity rose perpendicularly. What is more, the change was more obvious when the angle is raised. The rate of capacitance sensitivity change also showed the same regular pattern. 

In summary, to improve the sensitivity of the gyroscope, the spindle azimuth angle has to be increased. However, when the spindle azimuth angle approaches 90°, the gyroscope becomes nonlinear [35,36]. In addition, the capacitance changes rapidly and causes the gyroscope easily to be affected by fabrication imperfection. A small angular derivation can lead to a great change in the gyroscope’s sensitivity. Thus, keeping better robustness of the beam is the key in this research.

### 2.5. Fabrication Imperfection Analysis

From above, the spindle azimuth angle, can easily be affected by fabrication imperfection. Since fabrication technology is limited, there are fabrication imperfections at each step of fabrication process. This part is devoted to the influence of fabrication imperfections of different spindle azimuth angles in a convex cross-section of the oblique beam. Only the fabrication imperfections of the oblique beam’s cross-section are discussed; we assume that there are no fabrication imperfections in the other parts of the gyroscope.

**Figure 7:**
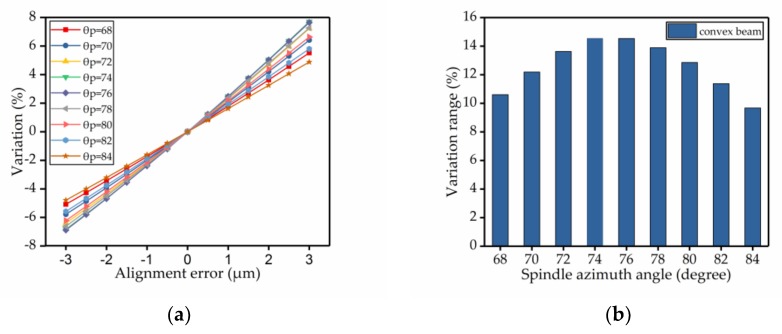
The influence of alignment errors on the spindle azimuth angle of the convex cross-section beam; (**a**) shows the relationship between the variation in the spindle azimuth angle and the alignment error; (**b**) shows the range in variation which is the absolute value of the difference between the maximum and minimum value of each spindle azimuth angle.

Based on the analysis of fabrication technology, before each step of fabrication process, photolithography must be adopted to get the structure of the figure. Thus, photolithography was repeated during the fabrication process. Therefore, the alignment error became the most common type of fabrication imperfection. So, we chose alignment error as an example and analyzed its effect on the spindle azimuth angle of the convex cross-section. Based on the fabrication process experience, we set the alignment error range from −3 μm to +3 μm. Then we analyzed the change between the actual value and the initially set value of the spindle azimuth angle, at different spindle azimuth angles, under the influence of alignment errors. As shown in Figure 7a, for each line, the initially set value of spindle azimuth angle was fixed. The line shows the trend for the variation in the spindle azimuth angle’s shift as the alignment error changed. Then, the absolute value of the difference between the maximum and the minimum value for each line was calculated. The result is shown in Figure 7b. It shows the maximum variation between different spindle azimuth angles for the whole range of alignment error. 

So, we found that the convex cross-section was influenced significantly by fabrication imperfections. This cross-section did not have good robustness. 

We found that the spindle azimuth angle needs to be sufficiently large to improve the sensitivity of a BFVG. Here, we propose a new polygonal cross-section shape which can adjust the spindle azimuth angle flexibly. This new cross-section was fabricated by AWE and DRIE-combined fabrication technology. It has better tolerance, thereby reducing the effect of fabrication imperfection on the spindle azimuth angle. The robustness of the BFVG’s structure is improved.

## 3. A Comparison and Analysis of the Tolerance of Different Cross-Sections

**Figure 8:**
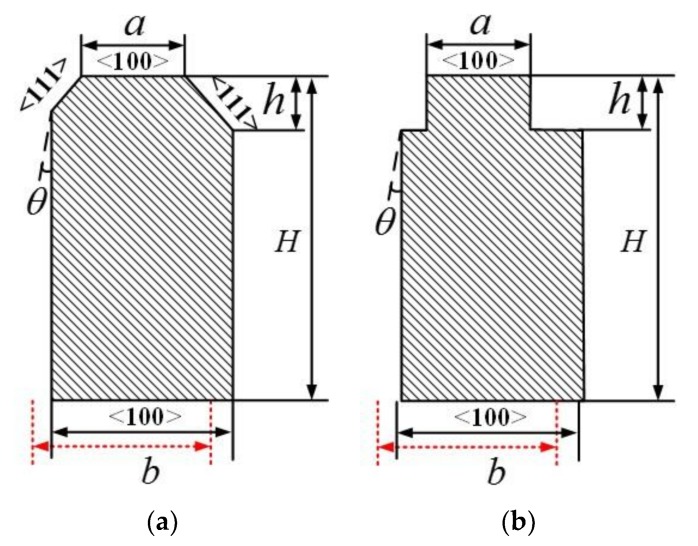
Fabrication imperfections of two kinds of oblique beam cross-section. (**a**) Polygonal cross-section; (**b**) convex cross-section.

Through the foregoing analysis, we can infer that there must have been some deviations during the fabrication of the gyroscope. The deviations will influence the sensitivity transformation of the BFVG. To make a comparison between the robustness of these two cross-sections, we considered the influence of fabrication imperfections for different spindle azimuth angles on polygonal and convex cross-sections of oblique beams. In this part, only the fabrication imperfections which affected the size of the cross-section are discussed. We assumed that there were no fabrication imperfections in the other parts of the gyroscope. The basic structure of the gyroscope is consistent to that described previously, except the oblique beam was tuned to the polygonal cross-section beam and the convex cross-section beam. The contrasts between these two types of cross-sections are shown in Figure 8. In order to compare the two, the initial rectangular beam was the same, and the cutting width (a) and the etching depth (h) had the same dimensions.

During the course of fabrication, the main fabrication imperfections in oblique beams include alignment errors, silicon thickness errors, etching depth errors, upper width errors, bottom width errors and DRIE verticality errors [37]. The different types of fabrication imperfections and the range of errors are listed in Table 2.

## 4. Tolerance Capability Analysis

**Figure 9:**
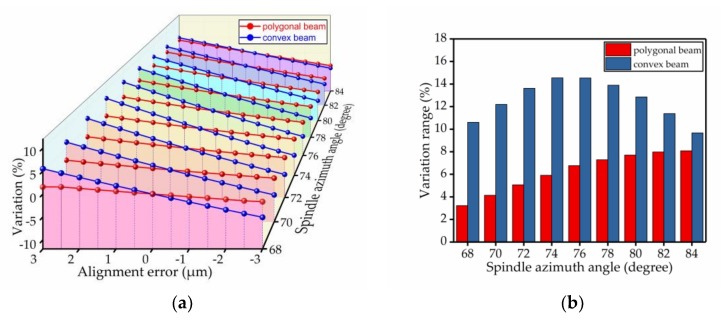
The influence of the alignment error on the spindle azimuth angle of the polygonal cross-section beam and convex cross-section beam; (**a**) shows the relationship between the spindle azimuth angle’s variation and the alignment error; (**b**) shows the range of variation—the value is the difference between the maximum and minimum value of each line.

**Figure 10:**
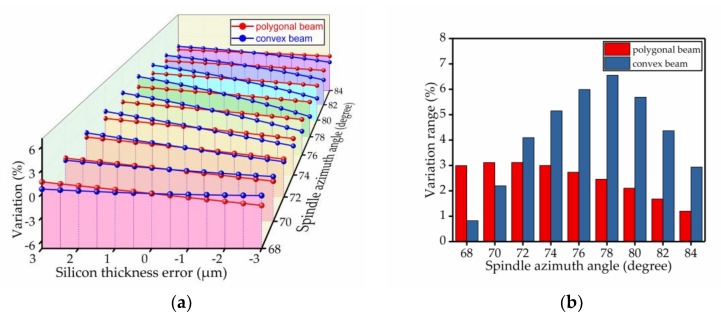
The influence of the silicon thickness error on the spindle azimuth angle of the polygonal cross-section beam and convex cross-section beam; (**a**) shows the relationship between spindle azimuth angle variation and silicon thickness error; (**b**) shows the range of variation—the value is the difference between the maximum and minimum values of each line.

**Figure 11:**
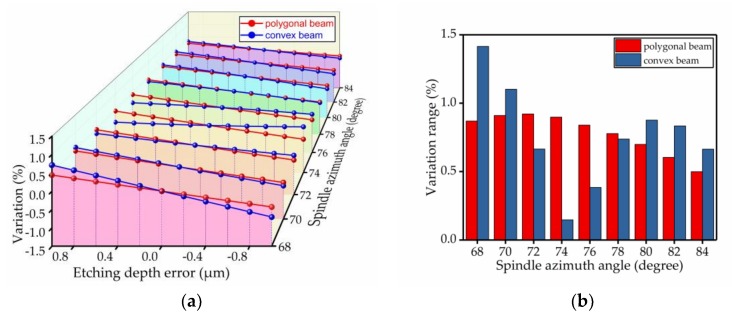
The influence of the etching depth error on the spindle azimuth angle of the polygonal cross-section beam and the convex cross-section beam; (**a**) shows the relationship between spindle azimuth angle variation and etching depth error; (**b**) shows the range of variation—the value is the difference between the maximum and minimum values of each line.

**Figure 12:**
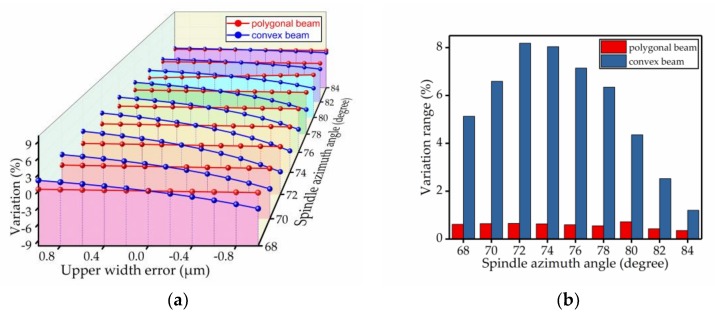
The influence of the upper width error on the spindle azimuth angle of the polygonal cross-section beam and convex cross-section beam; (**a**) shows the relationship between spindle azimuth angle variation and upper width error; (**b**) shows the range of variation—the value is the difference between the maximum and minimum values of each line.

**Figure 13:**
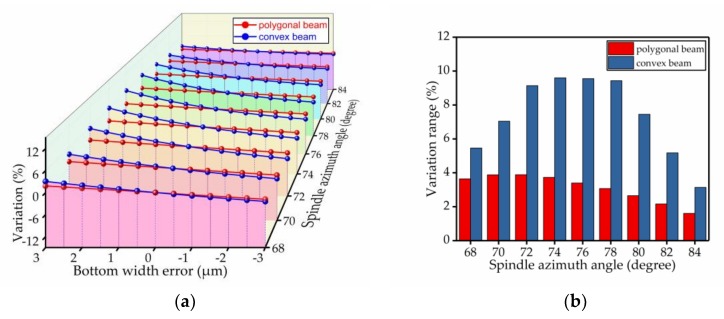
The influence of the bottom width error on the spindle azimuth angle of the polygonal cross-section beam and convex cross-section beam; (**a**) shows the relationship between spindle azimuth angle variation and bottom width error; (**b**) shows the range of variation—the value is the difference between the maximum and minimum values of each line.

**Figure 14:**
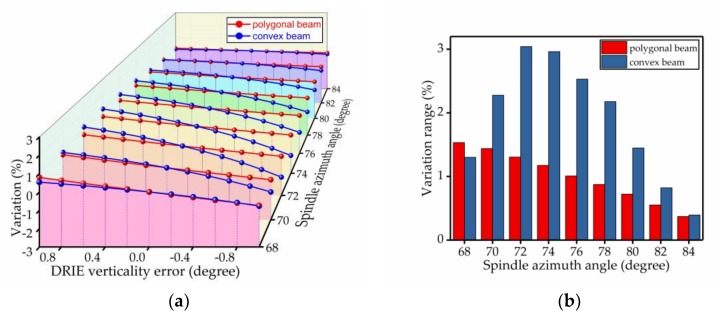
Influence of the DRIE verticality error on the spindle azimuth angle of the polygonal cross-section beam and convex cross-section beam; (**a**) shows the relationship between spindle azimuth angle variation and DRIE verticality error; (**b**) shows the range of variation—the value is the difference between the maximum and minimum values of each line.

The percentage change of the spindle azimuth angle was used to analyze the bias stability. The results of the comparison are shown in Figure 9, Figure 10, Figure 11, Figure 12, Figure 13 and Figure 14. Every figure includes two types of figures. The (a) type figure includes three axes, representing the spindle azimuth angle, fabrication imperfection and variation in the spindle azimuth angle. For each section, the abscissa coordinate shows the parameter date offset of different error types. The vertical coordinate shows the variation in the spindle azimuth angle caused by fabrication imperfection at a particular spindle azimuth angle. The extension coordinate shows the variation with different spindle azimuth angles. There are two comparison date curves in each spindle azimuth angle plane. The red curve represents the extent of change in the spindle azimuth angle of the polygonal cross-section beam with variation in dimensional deviation, and the blue curve represents the convex cross-section beam. The other (b) type figure includes two axes, representing the spindle azimuth angle and the variation in the spindle azimuth angle. The abscissa coordinate shows the spindle azimuth angle. The vertical coordinate shows the range of variation in the spindle azimuth angle caused by fabrication imperfection. The value was calculated from (a). It is equal to the difference between the maximum and minimum values of each line. The bar diagram shows the maximum variation range. The red bar represents the polygon beam, and the blue bar represents the convex cross-section beam.

### 4.1. Alignment Error

The alignment error refers to the size offset error present in the process of etching from the top silicon wafer to release the gyroscope structure after the Si–Si bonding, as shown by the red dashed line in Figure 8. During fabrication, the wafer is removed multiple times for a lithography process. Therefore, the alignment error is the most important fabrication imperfection that causes serious deviation away from the original design. By allowing for a certain amount of deviation, we compared the two cross-section beams in regard to the percentage change of the spindle azimuth angle. Ordinarily, the range of the alignment error deviation is from −3 μm to +3 μm. The figure shows that the percentage change of the polygonal cross-section beam is approximately half of the change in the convex cross-section beam, as shown in Figure 9.

### 4.2. Silicon Thickness Error

The silicon thickness error depends on the thickness error of the raw material, which is the silicon on insulator (SOI) silicon wafer. It is also an important error. The silicon thickness error is another important contributor to process deviations. We assumed that the other dimensions did not change and considered the thickness deviation of the silicon wafer, which was from −3 μm to +3 μm, at 0.5 μm intervals. Figure 10 shows that, in most cases, especially in the range of spindle azimuth angles that we needed, the influence of deviation on the polygonal cross-section beam was smaller than that on the convex cross-section beam, except for spindle azimuth angle between 68° and 70°, with silicon wafer thickness deviation. Thus, we conclude that the silicon thickness error’s effect on the polygonal cross-section is less than that of the convex cross-section.

### 4.3. Etching Depth Error

In addition, the percentage changes of the spindle azimuth angle for the two types of cross-section beams with the same etching depth error were characterized at different spindle azimuth angles, as shown in Figure 11. The deviation increased from −1 μm to +1 μm at 0.2 μm intervals, and each deviation is used to calculate the spindle azimuth angle for two cross-sections. The absolute value of the percentage change in the spindle azimuth angle with the polygonal oblique beam was similar to the convex oblique beam in the range from 68° to 72°. The influence of deviation on the polygonal cross-section beam was bigger than that on the convex cross-section beam in the range from 74° to 78°. This result presents a different phenomenon, whereby the influence of deviation on the polygonal cross-section beam is smaller than that on the convex cross-section beam when the spindle azimuth angle ranges from 80° to 84°.

The polygonal model was obtained by the AWE process and the convex cross-section beam was produced by the DRIE process. The AWE process has high controllability which can allow good control of the depth during corrosion. If we can achieve perfect alignment, there is non-existent etching depth deviation. However, the etching depth deviation always exists in DRIE processes and has a worse surface roughness than that of the AWE process.

### 4.4. Upper Width Error

Additionally, we further analyzed the impact of the upper width error. Ordinarily, the range of the upper width error deviation is from −3 μm to +3 μm at 0.5 μm intervals. The results from the date comparison are shown in Figure 12. The influence of deviation on the polygonal cross-section beam was smaller than on the convex cross-section beam. From Figure 12b, we can see that for different spindle azimuth angles, the upper width error’s influence on the convex cross-section was far greater than on the polygonal cross-section.

### 4.5. Bottom Width Error

We also analyzed the effect of the oblique beam’s bottom width. Based on the fabrication process experience, when we adopt dry-etching technology to release structure, the error caused by the technology ranges from about −3 μm to +3 μm. Here, we set the interval of error analysis to 0.5 μm. The results are shown in Figure 13. We found that the polygonal cross-section’s tolerance to the bottom width error was better than the convex cross-section’s tolerance.

### 4.6. DRIE Verticality Error

We know that the angle error caused by DRIE is unavoidable. The deeper the etching depth, the bigger the value of the DRIE verticality error and the greater the influence on the oblique beam’s spindle azimuth angle. Next, we set the DRIE verticality error range from −1° to +1°, with an interval of 0.2°, to analyze. The result shows that because the convex cross-section adopts dry-etching on the whole side wall and the side wall with unfilled corner, there is a DRIE verticality error. However, for the polygonal cross-section, the side wall with unfilled corner was processed by the AWE process; thus, the DRIE vertically error’s location was reduced. What is more, the angle error caused by the AWE process was minimal. This promoted the tolerance of the polygonal cross-section, as shown in Figure 14.

Overall, the polygonal oblique beam’s tolerance ability to fabrication imperfections is better than the convex oblique beam. Further, we obtained the range of spindle azimuth angles under different fabrication imperfections. The minimum and maximum values of the ranges are shown in Table 3. The convex cross-section beam is more sensitive to different fabrication imperfections. Under the influence of the fabrication imperfections, the robustness of polygonal cross-section was improved nine-fold at the best result and two-fold at the general results. It can be concluded that the polygonal cross-section has better robustness against fabrication imperfections.

## 5. Quadrature Analysis

The BFVG based on the Coriolis effect is equal to a second order mass-spring-damping system. Under theoretical conditions, its driving mode and sensing mode are completely orthogonal. Without the input of angular velocity, it vibrates periodically at a constant amplitude under the driving mode frequency and provides the initial velocity for detecting the Coriolis effect. Only when an angular velocity exists can the sensing mode can be motivated by Coriolis force. However, there are some factors that affect the performance of the gyroscope, such as material faults, fabrication imperfections, structural stress and so on. The trail of motion of the mass will be offset from the theoretical trail—the two operation modes of the BFVG are incompletely orthogonal. Thus, without the input of angular velocity, the detection circuit can detect the movement of the mass and produce the output with zero bias.

### 5.1. Simulation Model

Fabrication imperfections lead to a mechanical coupling error between the driving axis and the sensing axis. This produces the quadrature error. Thus, we compared the change in quadrature error between the polygonal oblique beam and the convex oblique beam under the same fabrication imperfections.

**Figure 15:**
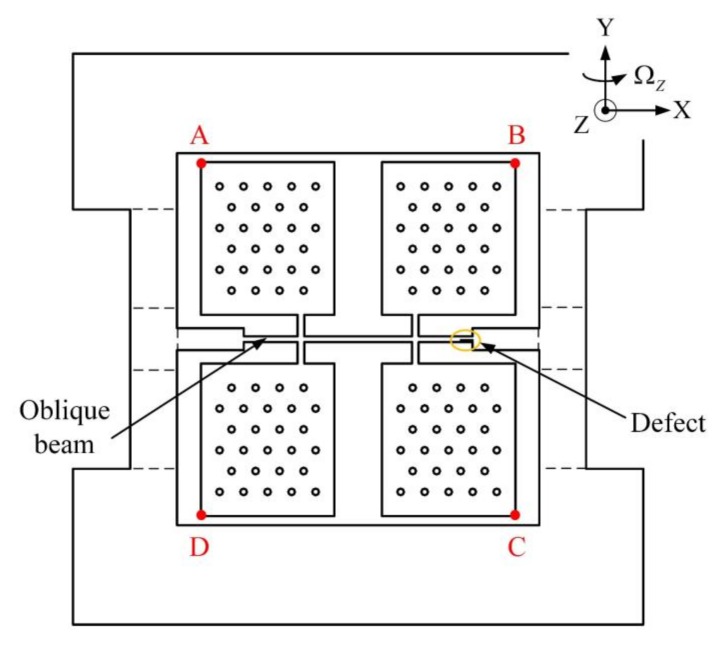
The sensitive structure of the BFVG: he four points with maximum amplitude are defined as A, B, C, D, and a fabrication defect is set near the anchor on the right side; the size of the fabrication defect is 50 μm × 10 μm × 100 μm.

Under the driving and sensing modes, we can use the change in displacement and the symmetry of displacement change for the designative points, at which the four masses achieve the maximum amplitude, to define the quadrature error caused by mechanical coupling error. Thus, the four points were set as A, B, C, D, as shown in Figure 15.

### 5.2. Comparison Date

We simulated the BFVG models with a polygonal cross-section beam and the convex cross-section beam with COMSOL. Then we set the same fabrication defect in the two models and compared the changes in displacement and symmetry of displacement change.

From above, the fabrication imperfections appeared to enhance the quadrature error. To compare the effect of these two cross-sections on the same fabrication defect, we first obtained the horizontal displacement and vertical displacement of the BFVG without defects under the driving mode. Then, we added a 50 μm × 10 μm × 100 μm cubic defect on the oblique beam in the two models and performed the same simulation. The change in displacement is shown in Table 4.

We found that the fabrication defect’s effect on the quadrature error was obvious. It not only confines the driving amplitude, but also reduced the efficiency of the gyroscope. When compared the change in displacement between the two cross-sections, the polygonal cross-section only had 34 percent (average value) of the change in the convex cross-section and the quadrature error caused by fabrication defect was smaller. This verifies the superiority of the polygonal cross-section.

What is more, we compared the symmetry of displacement change between the four points (A, B, C, D). From Section 2, we know that when the mass of BFVG is moving in the driving direction and sensing direction, the symmetric relationship between the four points is that shown in Table 5. In this paper, we define the symmetry of displacement change as follows: In different directions, the displacement ratio between the points shows a symmetric relationship. This data is shown in Table 5. We found that when there are fabrication defects on one side of the oblique beam, the degree of symmetry of displacement change of the four masses is better with the polygonal cross-section. This reduces the quadrature error of the gyroscope.

## 6. Conclusions

In this paper, we presented the analysis and design of a new oblique beam cross-section for BFVG. Based on the design model, the relationship between the spindle azimuth angle and sensitivity was analyzed concretely. We found that a larger spindle azimuth angle was associated with a more sensitive gyroscope. However, the spindle azimuth angle was easily affected by changes in the cross-section’s dimension. So, we had to design a strong, robust oblique beam cross-section which was flexible enough to be regulated at the spindle azimuth angle. The new polygonal cross-section was proposed. Then, we presented the effect of manufacturing deviations on different spindle azimuth angles which were suspended with the polygonal cross-section beam and convex cross-section beam. The percentage change in the spindle azimuth angle with the two types of cross-section beam was compared. Comparing multiple groups of results from different dates, the theoretical arithmetic results suggested that the polygonal cross-section beam is much more stable than the convex cross-section beam in most cases, especially when the spindle azimuth angle approaches 90°. Under the influence of the fabrication imperfections, the robustness of polygonal cross-section was improved nine-fold at the best result and two-fold at the general results. Additionally, we analyzed the quadrature error of these two cross-sections due to the fabrication defect’s effect. The quadrature error by fabrication defect was reduced by 70 percent with polygonal cross-section. Through theoretical analysis and simulation, we discovered that the polygonal cross-section had better robustness. Finally, because the sensitivity of the cross-section the beam to fabrication imperfections and the quadrature error was reduced, the consistency of fabrication process of the gyroscope was improved. The polygonal cross-section beam of the BFVG showed good validity and repeatability.

## Figures and Tables

**Table 1:** Structural parameters of a typical system (unit: μm).

Gyro Width	Gyro Height	Mass Width	Mass Height	Oblique Beam Width	Support Beam Width	Support Beam Height
$w_{0}$	$l_{0}$	$w_{1}$	$l_{2}$	$w_{2}$	$w_{3}$	$l_{1}$
6000	6000	1400	1600	60	70	220

**Table 2:** The range of fabrication imperfections.

Alignment Error	Silicon Thickness Error	Etching Depth Error	Upper Width Error	Bottom Width Error	DRIE Verticality Error
Red Dashed	H	h	a	b	θ
±3 μm	±3 μm	±1 μm	±1 μm	±3 μm	±1 deg

**Table 3:** The range of spindle azimuth angles under different fabrication imperfections (unit: %).

Error Types	Polygonal Cross-Section Beam 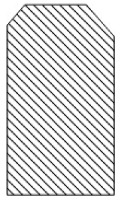	Convex Cross-Section Beam 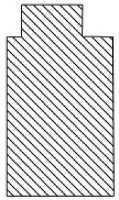
Alignment error	6.24089	12.5886
Silicon thickness error	2.48747	4.19936
Etching depth error	0.77974	0.75824
Upper width error	0.57613	5.50023
Bottom width error	3.11562	7.33358
DRIE verticality error	0.99716	1.88352

**Table 4:** The changes in displacement (unit: %).

Cross-Section Type	Displacement Direction	A	B	C	D
Polygonal cross-section beam	Driving direction (*X*)	0.078	0.016	1.051	0.754
	Sensing direction (*Z*)	10.725	4.467	10.113	5.514
Convex cross-section beam	Driving direction (*X*)	0.591	0.758	0.247	0.296
	Sensing direction (*Z*)	23.059	24.673	33.511	13.083

**Table 5:** The symmetry of displacement change (unit: %).

Cross-Section Type	Symmetry Point	Driving Direction (*X*)	Sensing Direction (*Z*)
Polygonal cross-section beam	A-B	1.118	*
	C-D	1.021	*
	A-C	*	1.534
	B-D	*	1.824
Convex cross-section beam	A-B	1.109	*
	C-D	1.05	*
	A-C	*	9.155
	B-D	*	2.039
